# Effect of decompressive laparotomy on organ function in patients with abdominal compartment syndrome: a systematic review and meta-analysis

**DOI:** 10.1186/s13054-018-2103-0

**Published:** 2018-07-25

**Authors:** Lana Van Damme, Jan J. De Waele

**Affiliations:** 1 Faculty of Medicine and Health Sciences, Ghent University, C. Heymanslaan 10, 9000 Ghent, Belgium; 2 Department of Critical Care Medicine, Ghent University Hospital, C. Heymanslaan 10, 9000 Ghent, Belgium

**Keywords:** Intensive care, Abdominal compartment syndrome, Decompressive laparotomy, Multiple organ failure, Hemodynamic recovery, Respiratory recovery, Kidney recovery, Mortality rate

## Abstract

**Background:**

Decompressive laparotomy has been advised as potential treatment for abdominal compartment syndrome (ACS) when medical management fails; yet, the effect on parameters of organ function differs markedly in the published literature. In this study, we sought to investigate the effect of decompressive laparotomy on intra-abdominal pressure and organ function in critically ill adult and pediatric patients with ACS, specifically focusing on hemodynamic, respiratory, and kidney function and outcome.

**Methods:**

A systematic review and meta-analysis of the literature was performed. Articles reporting data on intra-abdominal pressure (IAP), hemodynamic (mean arterial pressures [MAP], central venous pressure [CVP], cardiac index [CI], heart rate [HR], systemic vascular resistance index [SVRI] and/or pulmonary capillary wedge pressure [PCWP]), respiratory (positive end-expiratory pressure [PEEP], peak inspiratory pressure [PIP] and/or ratio of partial pressure arterial oxygen and fraction of inspired oxygen [P/F ratio]), and/or urinary output (UO) following decompressive laparotomy were analyzed.

**Results:**

A total of 15 articles were included; 3 included children only (aged 18 years or younger). Of the 286 patients who were included, 49.7% had primary ACS. The baseline mean IAP in adults decreased with an average of 18.2 ± 6.5 mmHg following decompression, from 31.7 ± 6.4 mmHg to 13.5 ± 3.0 mmHg. There was a decrease in HR (12.2 ± 9.5 beats/min; *p* = 0.04), CVP (4.6 ± 2.3 mmHg; *p* = 0.022), PCWP (5.8 ± 2.3 mmHg; *p* = 0.029), and PIP (10.1 ± 3.9 cmH_2_O; *p* < 0.001) and a mean increase in P/F ratio (70.4 ± 49.4; *p* < 0.001) and UO (95.3 ± 105.3 ml/h; *p* < 0.001). In children, there was a significant increase in MAP (20.0 ± 2.3 mmHg; *p* = 0.006), P/F ratio (238.2; *p* < 0.001), and UO (2.88 ± 0.64 ml/kg/h; *p* < 0.001) and a decrease in CVP (7 mmHg; *p* = 0.016) and PIP (9.9 cmH_2_O; *p* = 0.002). The overall mortality rate was 49.7% in adults and 60.8% in children following decompressive laparotomy.

**Conclusions:**

Decompressive laparotomy resulted in a significantly lower IAP and had beneficial effects on hemodynamic, respiratory, and renal parameters. Mortality after decompressive laparotomy remains high in both adults and children.

**Electronic supplementary material:**

The online version of this article (10.1186/s13054-018-2103-0) contains supplementary material, which is available to authorized users.

## Background

Abdominal compartment syndrome (ACS) is defined by an intra-abdominal pressure (IAP) of 20 mmHg or higher that is accompanied by newly developed organ dysfunction [1]. Although the first cases of ACS were described decades ago, interest has recently increased exponentially. Nevertheless, a recent survey demonstrated that knowledge among physicians is still suboptimal toward clinical management and awareness of ACS [2].

There is a wide range in the incidence of ACS in intensive care unit (ICU) patients, with patients in surgical ICUs more likely to develop ACS. Incidence rates of intra-abdominal hypertension (IAH) and ACS upon admission to the ICU are reported to be around 27.7% and 2.7%, respectively [3, 4]. The prevalence of ACS is between 4.2% and 14% in patients admitted to the ICU after trauma; in general ICUs, it is estimated to be around 1%. With increasing insight into the risk factors for ACS, the introduction of guidelines, and the availability of strategies to limit progression from IAH to ACS, ACS seems to be decreasing in most ICUs [5, 6].

As in other compartment syndromes, surgical decompression has been considered the definitive therapy for ACS for a long time, particularly in primary ACS. In case of secondary ACS, surgical intervention may no longer be the treatment of choice. The IAH/ACS management algorithm, as developed by WSACS (The Abdominal Compartment Society, formerly known as the World Society of the Abdominal Compartment Syndrome), was recently updated and recommends medical treatment options to reduce IAP before surgical decompression is needed [7, 8]. The cornerstone of medical management in patients with IAH (defined as IAP ≥ 12 mmHg) is perfusion support and optimized fluid management, with several noninvasive methods used to reduce IAP, such as nasogastric decompression or percutaneous drainage of fluid collections. Only when these noninvasive medical treatment options fail to lower the IAP and organ failure persists is decompressive laparotomy recommended [1, 9–11].

The effect of decompressive laparotomy on organ function in patients with ACS has been poorly described. Because mortality rates remain as high as 49% even after decompression [12], further investigation is needed into the use of decompressive laparotomy to understand who could benefit the most. From a mechanistic point of view, for example, patients with decreased abdominal wall compliance would benefit the most, but this has been largely ignored. Our goal in this study was to investigate the effect of decompressive laparotomy on organ function, particularly on hemodynamics and respiratory and renal function, in a broad ICU population.

## Methods

### Search strategy

The literature was reviewed for studies reporting on the effect of decompressive laparotomy in patients with ACS published between 1995 and September 2017. The search terms (“abdominal compartment syndrome” or “intra-abdominal hypertension” or “intra-abdominal pressure” and “decompression” or “decompressive surgery” or “decompressive laparotomy”) were used to search three databases (MEDLINE, Embase, and Web of Science). Restrictions were applied to the search query. To minimize publication bias, case reports were excluded, as were case series or reviews describing fewer than five patients. In addition, animal studies were not considered. The search was limited to literature published in the English, French, German, Dutch, and Spanish languages. The bibliographies of the included articles were examined for relevant publications that might have been overlooked otherwise.

### Inclusion and exclusion criteria

Eligible studies were assessed on the basis of predefined inclusion and exclusion criteria. Studies that described adult and pediatric patients who developed ACS and required decompressive laparotomy were included in the analysis.

Studies were included only if they reported the IAP at least before the procedure, or if they defined ACS, in addition to patient outcome for every patient who underwent decompressive laparotomy. Hemodynamic (blood pressure [BP], heart rate [HR], systemic vascular resistance index [SVRI], cardiac index [CI], pulmonary capillary wedge pressure [PCWP], and/or central venous pressure [CVP]), renal (urinary output [UO]), and/or respiratory (ratio of partial pressure arterial oxygen and fraction of inspired oxygen [P/F ratio], peak expiratory end pressure [PEEP], and/or peak inspiratory pressure [PIP]) parameters were required to be reported to measure the effect on organ dysfunction. Patients who underwent decompressive laparotomy needed to be clearly identified, and the data of these patients had to be discussed separately. The time frame of measuring these parameters had to be less than 72 hours after the intervention.

Decompressive laparotomy was defined as a vertical, midline, full-thickness abdominal incision aimed at reducing the IAP. This may or may not have been followed by a temporary abdominal closure. Other decompression techniques, such as subcutaneous fasciotomy or subcostal laparotomy, were not included.

### Data extraction and outcome measures

Baseline characteristics of the patients as well as of the studies were extracted. These included first author; year of publication; number of patients; study design; and basic patient characteristics, such as age, gender, and cause of ACS. Studies were categorized according to whether the WSACS definition [1] was used in the diagnosis of ACS. Each article was then categorized according to which type of ACS it described, namely primary, secondary, or combined. Studies reporting pediatric patients were analyzed separately. Hemodynamic, renal, and/or respiratory parameters were extracted and analyzed.

### Statistical analysis

For statistical analysis, the Comprehensive Meta-Analysis software package (Biostat, Englewood, NJ, USA) was used. The outcome parameters retrieved from the studies (described as mean, SD, and sample size) were entered into the program to calculate the standardized mean difference (SMD) of each parameter as well as the *p* value. When articles described median and range, an estimation of mean and SD was made using the formula described by Hozo et al. [13]. Heterogeneity across studies was evaluated using Cochran’s *Q* statistic. A random effects model was used when heterogeneity was present, as suggested by DerSimonian and Laird, to reduce bias [14]. A *p* value < 0.1 for the Cochran’s *Q* statistic was considered to represent significant between-study heterogeneity. The *p* value was considered statistically significant when this was < 0.05. Hedges’ *g* was used to examine the SMD, because the *p* value provides information about only the presence of an effect and not the size of the effect. The following cutoffs were considered to estimate the effect size of the intervention on the reported parameter: 0.2–0.5, a small effect size; 0.5–0.8, a medium effect size; and > 0.8, a large effect size.

### Quality assessment

A funnel plot was used to assess the presence of publication bias visually and was quantified by the Egger test. The threshold for bias was a *p* value < 0.10. Two validated checklists were conducted to assess the methodological quality of all included studies. First, the methodological index for nonrandomized studies (MINORS) [15] was used, which contained a checklist consisting of 8 criteria for noncomparative studies and 12 items for comparative studies. Downs and Black [16] drafted a checklist for not only nonrandomized but also randomized studies. With this list, 27 items were verified to assess the quality of the studies. To facilitate assessment, combining both studies [32, 33], each score was converted to a 0–10 scale, and an average score was then calculated. The lower the score, the higher the risk of bias, and vice versa.

## Results

### Study flow and characteristics

**Fig. 1:**
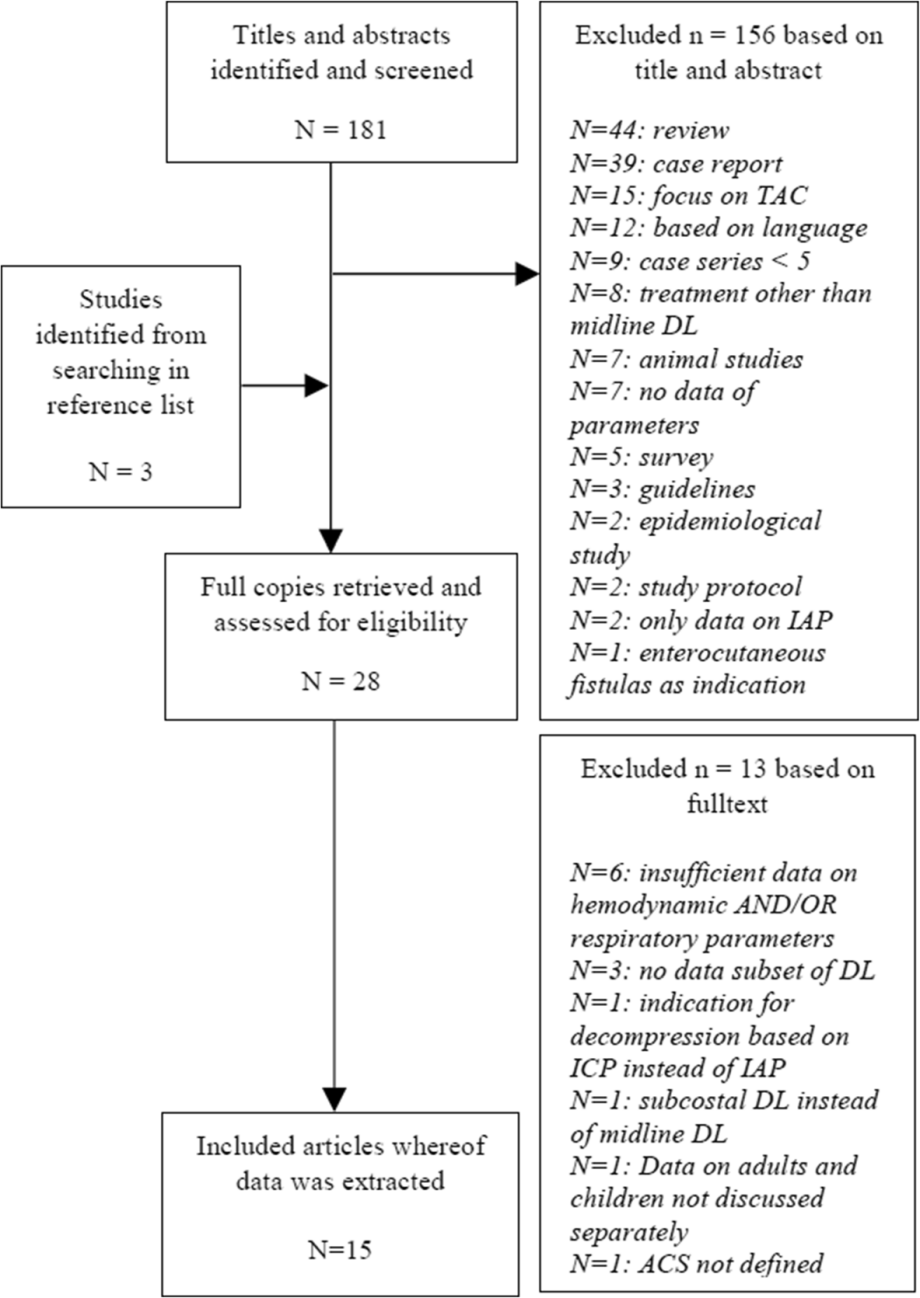
Study selection flowchart

The literature search identified 184 articles, which were retrieved for further evaluation. Of these, 156 studies were excluded on the basis of title and abstract, and of the 28 full-text articles reviewed, 13 were excluded because the necessary information was lacking. Ultimately, 15 articles were included in the analysis. A summary of the study identification and selection flow is provided in Fig. 1.

Overall, 286 patients with 292 ACS episodes were included in the analysis. Three articles reported on patients aged 18 years or younger. Of the 286 patients who were included, 49.7% had primary ACS. The study characteristics are described in Table 1. Seven of the included articles described prospective observational cohort studies, six reported retrospective observational cohort studies, one was a case series, and another one combined both prospective and retrospective data.

**Table 1:** Baseline study and patient characteristicsᅟ

First author [reference]	Year	Study design	No. of patients	Primary ACS	Secondary ACS	M/F	Baseline IAP^a^	Time to decompress	Mortality rate	Definition ACS
Meldrum et al. [21]	1997	Prospective study	21	21	0	15/6	27 ± 2.3	27 ± 4 hours	28.6%	Grades III and IV
Chang et al. [22]	1998	Prospective study	11	11	0	8/3	/	38 ± 34 hours	63.6%	Grade IV
Ertel et al. [23]	2000	Prospective and retrospective	17	17	0	14/3	42 ± 20.6	12.9 ± 2 hours	35.3%	Grade IV
Kopelman et al. [24]	2000	Case series	6	0	6	6/0	41.5 ± 11.5	/	66.7%	Grade IV
Beck et al.^b^ [18]	2001	Prospective study	10 (15^c^)	0	10	/	23.9 ± 3.8	/	60%	Grades II, III, and IV
Mcnelis et al. [25]	2002	Retrospective study	18 (19^c^)	3	15	6/12	43.4	/	61.1%	Grade IV
Mayberry et al. [26]	2003	Retrospective study	9	0	9	6/3	36 ± 5	17 ± 15 hours	22.2%	Grades III and IV
Balogh et al. [27]	2003	Prospective study	26	11	15	20/6	36 ± 15.3	13 ± 2 hours	57.7%	Grade IV
Dolores-Velasquez et al. [28]	2003	Prospective study	10	0	10	7/3	30.2 ± 7.6	/	30%	Grade IV
Zhou et al. [29]	2010	Retrospective study	16	4	12	6/10	25.2 ± 3.6	26 ± 11.2 hours	/	Grades III and IV
Struck et al. [30]	2013	Retrospective study	35	0	35	24/11	33 ± 7	/	71.4%	Grades III and IV
Rollins et al.^2^ [31]	2013	Retrospective study	7	0	7	6/1	28.3 ± 6.7	/	100%	Grades II, III, and IV
Divarci et al.^2^ [17]	2014	Prospective study	6	2	4	/	20.7 ± 3	/	16.7%	Grades II, III, and IV
De Waele et al. [32]	2016	Prospective study	33	27	6	20/13	23 ± 1.5	/	36.4%	Grades III and IV
Peng et al. [33]	2016	Retrospective study	61^d^	48	10	35/26	34 ± 7.75	64 ± 22.5	52.5%	Grades III and IV

*ACS* Abdominal compartment syndrome, *IAP* Intra-abdominal pressure, / = not available

^a^Intra-abdominal pressure before decompressive laparotomy (in mean ± SD)

^b^Articles describing children

^c^Total number of episodes

^d^Etiology unknown in three patients

### Publication bias and sensitivity analysis

**Fig. 2:**
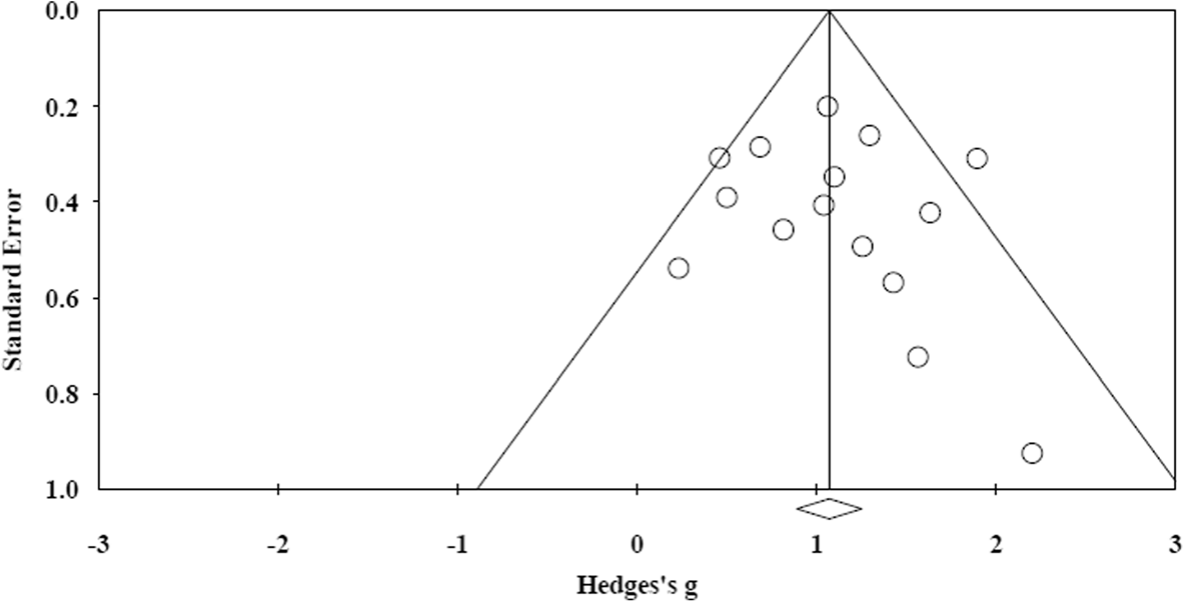
Funnel plot of all articles included in this study

The study funnel plot showed a quite symmetrical dispersion of the studies, as can be seen in Fig. 2. When the results were quantified through an Egger’s regression intercept, a *p* value (one-sided) of 0.33 was calculated. The methodological quality of the included studies, based on the validated checklists, showed two studies with low scores, six with moderate scores, another five with moderate to high scores, and two with high scores. The scoring of the assessment is provided in Table 2*.*

**Table 2:** Methodological quality scores based on MINORS and Downs and Black

First author [reference]	MINORS	0–10	Downs and Black	0–10	Mean
Meldrum et al. [21]	9	5.6	16	5	5.3
Chang et al. [22]	8	5	18	5.6	5.3
Ertel et al. [23]	10	6.3	23	7.2	6.8
Kopelman et al. [24]	6	3.8	9	2.8	3.3
Beck et al. [18]	9	5.6	17	5.3	5.5
Mcnelis et al. [25]	9	5.6	20	6.3	6.7
Mayberry et al. [26]	10	6.3	22	8.1	7.2
Balogh et al. [27]	12	7.5	20	6.3	6.9
Dolores-Velasquez et al. [28]	12	7.5	21	6.6	7.1
Zhou et al. [29]	10	6.3	15	4.7	5.5
Struck et al. [30]	8	5	19	5.9	5.5
Rollins et al. [31]	5	3.1	13	4.1	3.6
Divarci et al. [17]	10	6.3	17	5.3	5.8
De Waele et al. [32]	11	6.9	21	6.6	6.8
Peng et al. [33]	11	6.9	20	6.3	6.6

*MINORS* Methodological index for nonrandomized studies

### Effect of decompressive laparotomy in adults

#### Effect of decompressive laparotomy on IAP in adults

**Fig. 3:**
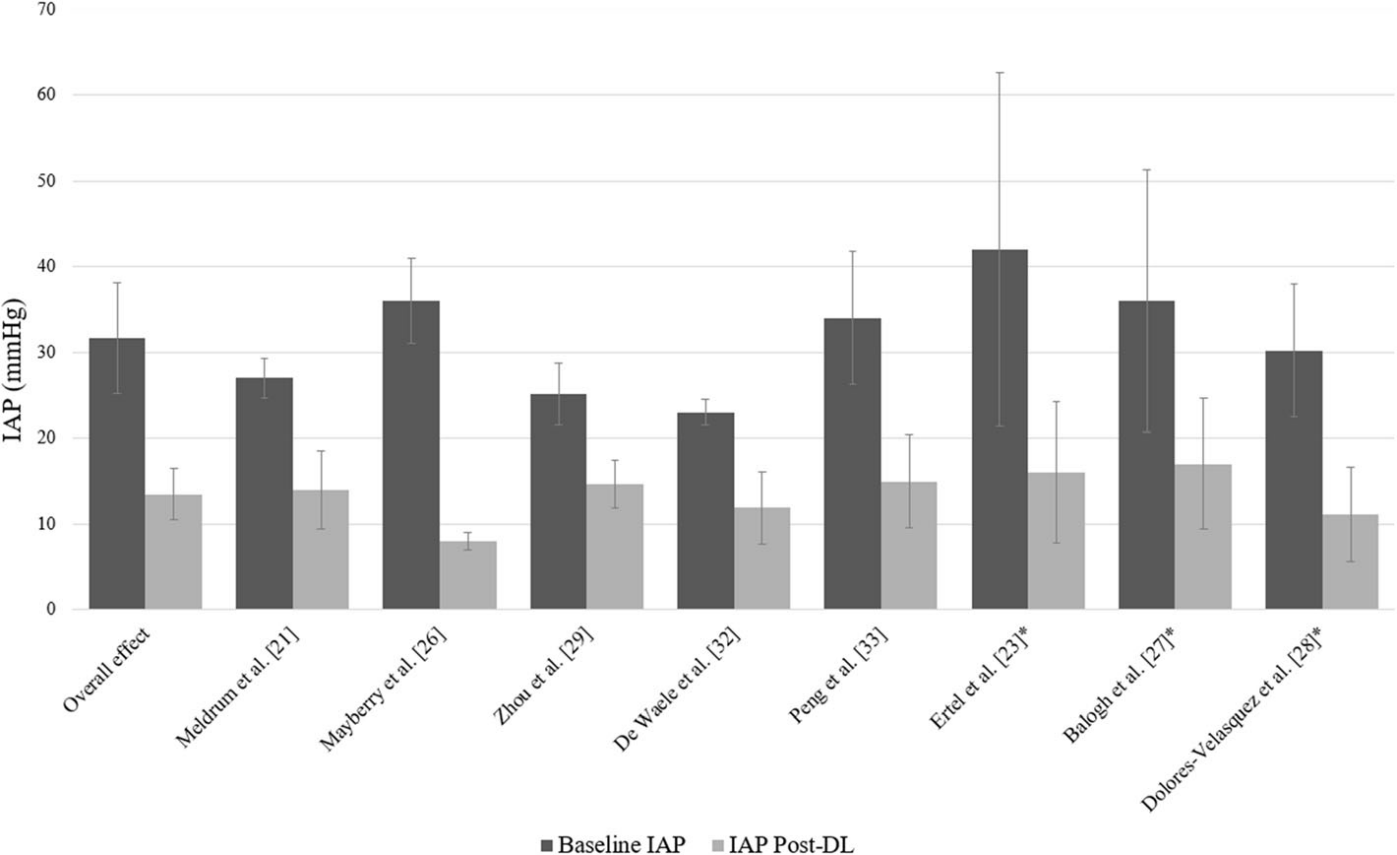
Effect of decompressive laparotomy on IAP in adult patients. The baseline intra-abdominal pressure is shown as a dark gray column. The light gray column represents the postoperative intra-abdominal pressure. *Articles reporting on grade IV ACS. *IAP* Intra-abdominal pressure, *DL* Decompressive laparotomy

The effect of decompressive laparotomy on IAP was available in 8 of 15 studies and is summarized in Fig. 3*.* The baseline mean IAP was 31.7 mmHg and ranged from 23 mmHg to 43.4 mmHg. Following decompression, the IAP decreased to an average of 13.5 mmHg, varying between 11 and 17 mmHg.

#### Effect on hemodynamics, the respiratory system, and kidney function in adults

The effect on the different organ systems is summarized in Table 3*.* Regarding hemodynamics, the largest SMD (1.117) was observed for PCWP, where there was a mean overall decrease of 5.8 ± 5 mmHg after decompressive laparotomy; the CVP decreased by 4.6 ± 2.3 mmHg. The overall effect on respiratory function in adults was available in 9 of 12 studies. For the PIP, there was a mean decrease of 10.1 ± 3.9 cmH_2_O following decompressive laparotomy. The P/F ratio increased with 70.4 ± 49.4 mmHg postdecompression. The effect on UO was reported in 197 patients; an overall mean increase of 95.3 ± 105.3 ml/h was reported after decompressive laparotomy.

**Table 3:** Effect of decompressive laparotomy on organ function in adults (all patients)

Variable	No. of studies reporting data of variables	Mean preoperative value	Mean postoperative value	Mean difference following DL	Heterogeneity			Hedges’ *g*	*p* Value
					Cochran’s *Q*	*df*	*p* Value		
IAP (mmHg)	8	31.7 ± 6.4	13.5 ± 3.0	− 18.2 ± 6.5	56.373	7	< 0.001	2.222	< 0.001
HR (beats/min)	4	122 ± 10.6	109 ± 11.6	− 12.2 ± 9.5	7.623	3	0.054	0.505	0.040
MAP (mmHg)	5	82 ± 16.3	88.8 ± 21.7	+ 6.8 ± 15.7	40.398	4	< 0.001	0.173	0.698
CVP (mmHg)	6	18.6 ± 3.1	14 ± 2.9	− 4.6 ± 2.3	13.030	5	0.023	0.624	0.022
PCWP (mmHg)	5	23.7 ± 6.7	17.9 ± 3.7	− 5.8 ± 5	41.008	4	< 0.001	1.117	0.029
CI (L/min/m^2^)	6	4 ± 1.2	4.6 ± 1.5	+ 0.82 ± 0.8	8.145	5	0.148	0.569	0.002
SVRI (dyn·s/cm^5^·m^2^)	3	2009.3 ± 364.5	1484 ± 451.2	311.3 ± 539.4	42.86	2	< 0.001	0.534	0.28
PIP (cmH_2_O)	9	45.5 ± 8.7	35.6 ± 8.0	− 10.1 ± 3.9	22.310	8	0.004	1.344	< 0.001
PEEP (cmH_2_O)	2	18.2 ± 13.1	17.4 ± 13.2	− 0.9 ± 0.1	0.002	1	0.966	0.078	0.768
P/F ratio	8	163.7 ± 48.4	234.1 ± 55.8	+ 70.4 ± 49.4	11.889	7	0.104	0.894	< 0.001
UO (ml/h)	8	44.1 ± 30.0	139.4 ± 109.4	+ 95.3 ± 105.3	23.687	7	0.001	1.061	< 0.001

*Abbreviations: IAP* Intra-abdominal pressure, *HR* Heart rate, *MAP* Mean arterial pressure, *CVP* Central venous pressure, *PCWP* Pulmonary capillary wedge pressure, *CI* Cardiac index, *SVRI* Systemic vascular resistance index, *PIP* Peak inspiratory pressure, *PEEP* Positive end-expiratory pressure, *P/F ratio* Ratio of partial pressure arterial oxygen and fraction of inspired oxygen, *UO* Urinary output

#### Effect of decompressive laparotomy in patients with grade III/IV and grade IV ACS

Five studies described patients with grades III and IV ACS. In these patients, the mean baseline IAP was 29 mmHg (23–36 mmHg). The mean postoperative IAP was 12.7 mmHg (8–15 mmHg). For the articles reporting grade IV ACS, the mean preoperative IAP was 36.1 mmHg (30.2–42 mmHg). After decompressive laparotomy, the IAP decreased to a mean value of 14.7 mmHg (11.1–17 mmHg).

The effect on hemodynamic, respiratory, and kidney function parameters is summarized in Additional files 1 and 2. In the studies on grades III and IV ACS, the mortality rate was 42% (22.2–71.4%). In grade IV ACS, there was a mortality rate of 52% (30–61.1%).

#### Outcome after decompressive laparotomy in adult patients

**Fig. 4:**
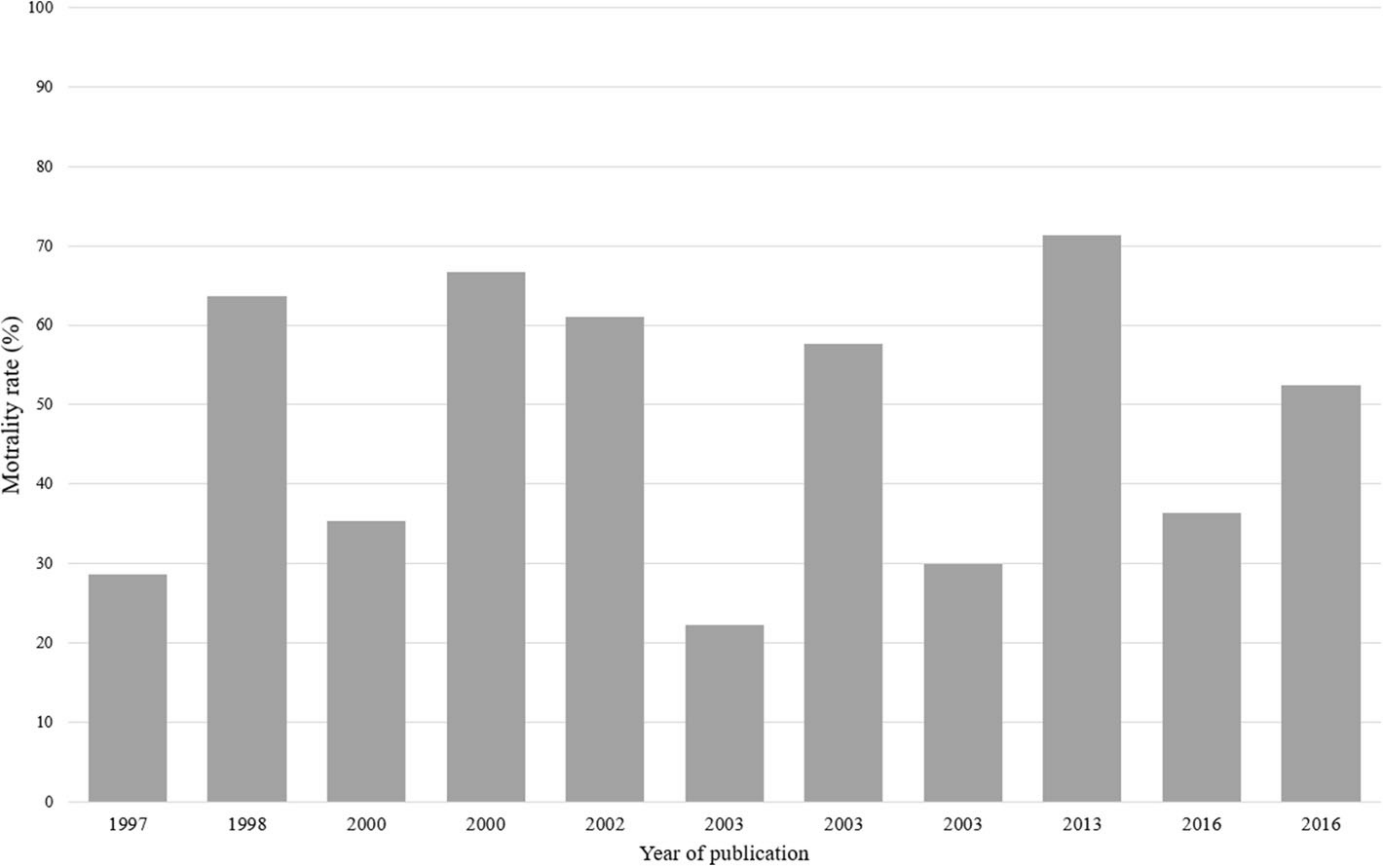
Effect of decompressive laparotomy on mortality rate in adults, according to year of publication

**Fig. 5:**
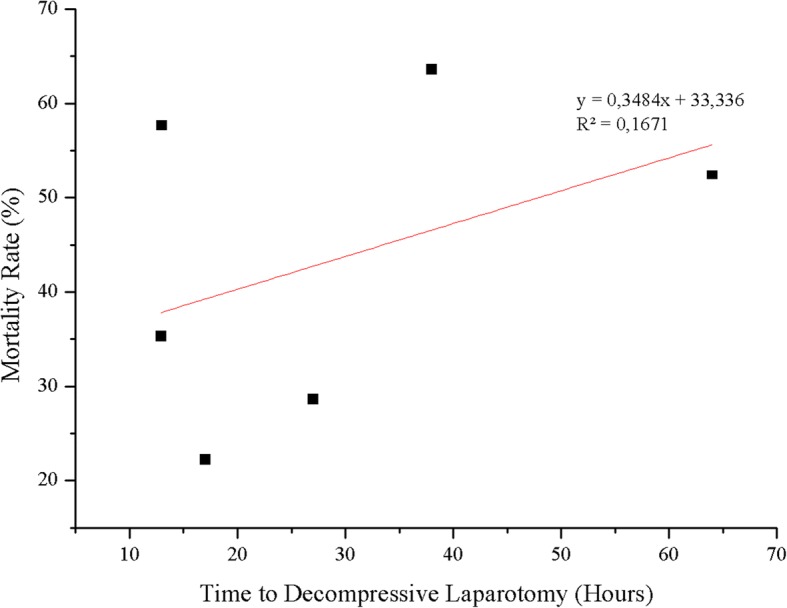
Time to decompressive laparotomy and mortality rate in adults

The mortality rate was reported in 11 of 12 studies and ranged from 22.2% to 71.4% in the individual studies; overall mortality was 49.7% (123 of 247 patients died). Of the articles that reported the mortality rate, the cause of death was described in 46 patients. Multiple organ failure was the main cause of death, followed by intestinal ischemia or necrosis. The results are summarized in Table 4*.* As can be seen in Fig. 4, no trend over time was observed regarding mortality rate following decompressive laparotomy. There was a trend toward a statistically significant correlation between time to decompression and mortality (grades III and IV *p* = 0.01, *F* = 30.75, *R*^2^ = 1). However, a nonsignificant correlation between time to decompression and mortality rate was found when all adult patients were considered (*R*^2^ = 0.167, *F* = 0.803; *p* = 0.065) (Fig. 5).

**Table 4:** Cause of death in adults undergoing decompressive laparotomy

**Organ failure**	**41**
▪ Multiple organ failure	30
▪ Intestinal ischemia and/or necrosis	7
▪ Respiratory failure	3
▪ Cardiac failure	1
**Other causes**	**5**
▪ Hemorrhagic shock	2
▪ Septic shock	2
▪ Severe head injury	1
**Not reported**	**77**

Bold data represents the overall number of deaths following DL. A subdivision is given to specify the cause

### Effect of decompressive laparotomy in children

#### Effect of decompressive laparotomy on IAP in children

**Fig. 6:**
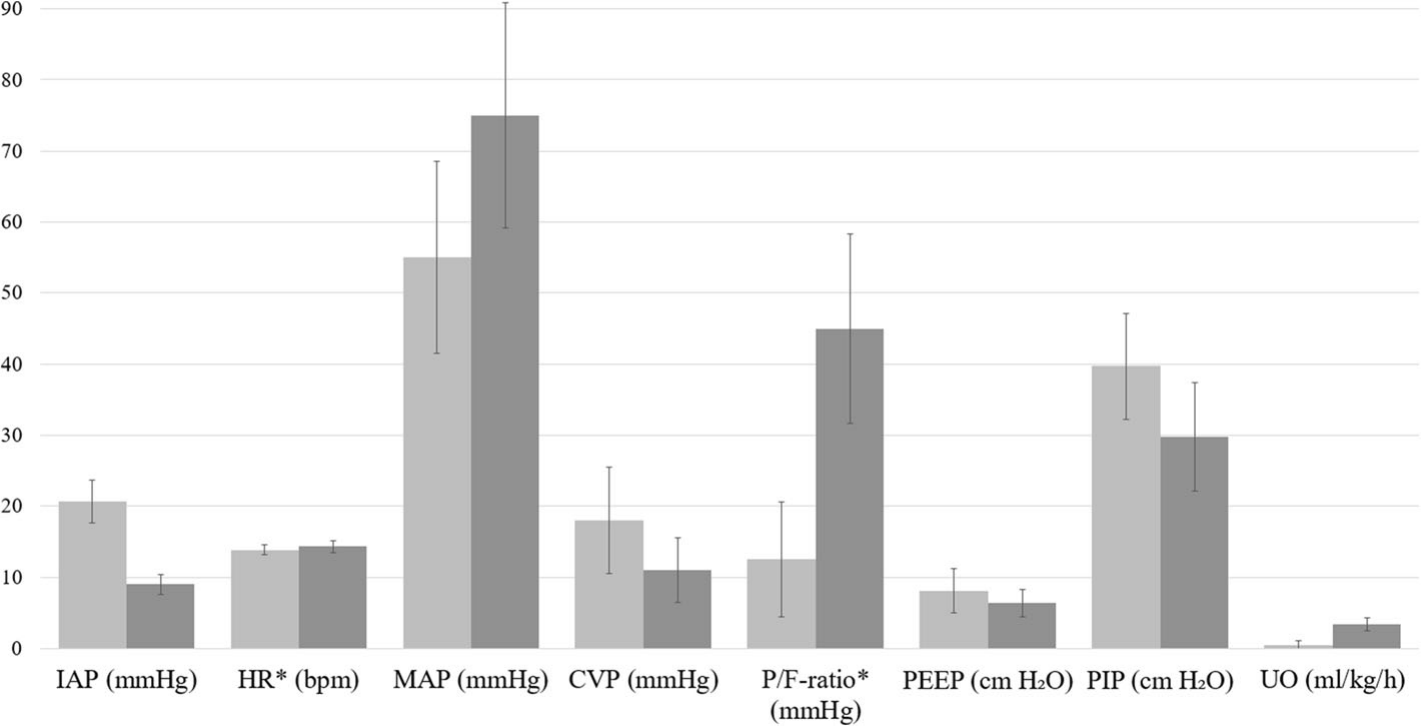
Effect of decompressive laparotomy on organ function parameters in children. Preoperative IAP is shown as a light gray column. The dark gray column represents the postoperative intra-abdominal pressure. *HR and P/F ratio are shown as one-tenth of actual value. *IAP* Intra-abdominal pressure, *HR* Heart rate, *MAP* Mean arterial pressure, *CVP* Central venous pressure, *PIP* Peak inspiratory pressure, *PEEP* Positive end-expiratory pressure, *P/F ratio* Ratio of partial pressure arterial oxygen and fraction of inspired oxygen, *UO* Urinary output

In children, only one article [17] reported both baseline and postdecompression values of IAP. At the time of decompression, the IAP was 20.7 mmHg and decreased to 9 mmHg thereafter (*p* < 0.001; SMD = 4.614) (Fig. 6).

#### Effect on hemodynamics, the respiratory system, and kidney function in children

The effect on HR was reported in 16 children. There was a mean baseline HR of 138.4 beats/min that increased by 4.4 ± 1.3 beats/min after decompression (*p* = 0.642; SMD = − 0.156). The MAP was reported in the same group and increased with 19.96 ± 2.34 mmHg following decompressive laparotomy (*p* = 0.006; SMD = 1.139). One article reported the effect on CVP in seven children and showed a decrease following decompressive laparotomy from a baseline of 18 mmHg to 11 mmHg (*p* = 0.016; SMD = 1.354). There was only one article [18] reporting the effect on respiratory parameters in children, describing a population of ten children. The baseline P/F ratio of 125.7 increased to 449.6 following decompressive laparotomy (*p* < 0.001; SMD = 2.818). In PEEP, there was a decrease following decompression from 8.1 cmH_2_O to 6.3 cmH_2_O (*p* = 0.129; SMD = 0.671). A baseline PIP of 39.7 cmH_2_O was reported and decreased to a postdecompression pressure of 29.8 cmH_2_O (*p* = 0.002; SMD = 1.264). All three articles describing children reported the effect on UO, providing data on a total of 23 children. There was a baseline output of 0.52 ml/kg/h, ranging from 0.02 to 1.1 ml/kg/h, that increased to 3.4 ml/kg/h, varying between 2.3 and 4.0 ml/kg/h (*p* < 0.001; SMD = 1.363).

#### Outcome after decompressive laparotomy in children

In children requiring decompressive laparotomy, the mortality rate was 60.8% (16.7–100%); 14 of 23 children died. The cause of death was described in 10 of the 14 children; these data can be found in Table 5.

**Table 5:** Cause of death in children undergoing decompressive laparotomy

**Organ failure**	**3**
▪ Multiple organ failure	2
▪ Cardiac failure	1
**Other causes**	**7**
▪ Brain death whilst on ECMO	5
▪ Mechanical bowel obstruction	1
▪ Seizures resulting in brain death	1
**Not reported**	**4**

Bold data represents the overall number of deaths following DL. A subdivision is given to specify the cause

## Discussion

In this systematic review and meta-analysis, we found that decompressive laparotomy results in a significant decrease in IAP. Decompressive laparotomy also had a measurable effect on organ failure, especially on respiratory function as well as on kidney function. There was a small effect on hemodynamics, which could be seen mainly in grade IV ACS. Mortality remains high; 49.7% of adults did not survive, underlining the severity of the illness. In children, the mean mortality rate was as high as 60.8% following decompressive laparotomy.

When laparotomy is performed, most hemodynamic, respiratory, and renal parameters will improve. Therefore, the results of this meta-analysis confirm the recommendation that decompressive laparotomy should be considered when medical options fail. Clearly, though, there is still room for improvement, and new options should be explored. It is incompletely understood which patients would benefit most from decompressive laparotomy or what is the most optimal timing for the intervention. As demonstrated in this analysis, there was a correlation between timing of decompressive laparotomy and mortality, although the correlation was weak and the clinical relevance limited. However, it is worth mentioning that in the studies in this analysis, some centers may have been reluctant to perform decompressive laparotomy, whereas others operated more rapidly, potentially leading to bias. Furthermore, we did not have individual-patient data available. When considering who would be the ideal candidates for decompressive laparotomy, it can be assumed that patients with low abdominal wall compliance are most likely to develop ACS and would probably benefit most from decompressive laparotomy. If there were methods available to easily identify these patients, a more individualized treatment approach would be possible [19, 20].

Overall, organ failure is poorly defined in the reviewed articles, and many studies fail to report the true markers of organ failure. The use of the Sequential Organ Failure Assessment (SOFA) scoring system has been recommended before, in the WSACS guidelines of 2013 [1]; the two most recent studies that were included in the present review did report SOFA before as well as after decompression. However, the SOFA scoring system has its own limitations. Minor changes (in any of the organ systems) that can be beneficial in patients might be overlooked because of the broad range within gradation scores; for example, the P/F ratio may increase from 110 to 190, but still the SOFA score would remain unchanged.

A positive trend can be seen in the baseline IAP, where pressures over 40 mmHg have become rare because IAP measurement is now more frequent and IAH and ACS are detected earlier. Even though there is a lower baseline IAP, the mortality rate did not improve over the years. Mortality was higher in grade IV ACS (52%) than in grades III and IV ACS combined (42%).

Because of the detrimental effects of ACS and the need for well-researched therapeutic options, articles need to describe not only IAP but also hemodynamic, respiratory, and renal parameters. Measuring protocols should be standardized to allow for a more complete description of data. Currently, different scoring systems and parameters are used to describe the results regarding multiple organ failure, whereas when describing outcome, to get a better understanding of the effect of an intervention, uniformity is key. Because mortality remains high, it is of the essence to report the cause of death when possible.

This study has several limitations. Before all the data could be analyzed, some of the data had to be converted from SEM to SD. Bessel’s correction was used to correct bias in the estimation of the population variance. Nevertheless, the mean SE will often be raised in these approximations, and therefore results could vary. Furthermore, some articles reported only median and range data. The formula described by Hozo et al. to convert median values to mean and SD was used [13]. This might have affected outcomes, specifically in variance. Nonetheless, the alternative of not using these articles might be less favorable than using the estimated mean.

## Conclusions

Decompressive laparotomy results in a significantly lower IAP in both adults and children with ACS. There was an improvement in hemodynamics, but these changes were not as substantial as observed in respiratory and renal function parameters. Even when ACS is treated, mortality remains high because one of two adult patients died after decompressive laparotomy. Better patient selection and optimized timing of the intervention may result in better clinical outcomes in the future.

## Additional files

Additional file 1: Effect of decompressive laparotomy on organ function in adults with grades III and IV ACS. *IAP* Intra-abdominal pressure, *HR* Heart rate, *MAP* Mean arterial pressure, *CVP* Central venous pressure, *PCWP* Pulmonary capillary wedge pressure, *CI* Cardiac index, *PIP* Peak inspiratory pressure, *PEEP* Positive end-expiratory pressure, *P/F ratio* Ratio of partial pressure arterial oxygen and fraction of inspired oxygen, *UO* Urinary output. (XLSX 10 kb)

Additional file 2: Effect of decompressive laparotomy on organ function in adults with grade IV ACS. *IAP* Intra-abdominal pressure, *HR* Heart rate, *MAP* Mean arterial pressure, *CVP* Central venous pressure, *PCWP* Pulmonary capillary wedge pressure, *CI* Cardiac index, *SVRI* Systemic vascular resistance index, *PIP* Peak inspiratory pressure, *PEEP* Positive end-expiratory pressure, *P/F ratio* Ratio of partial pressure arterial oxygen and fraction of inspired oxygen, *UO* Urinary output. (XLSX 11 kb)

## Abbreviations

ACS: Abdominal compartment syndrome
BP: Blood pressure
CI: Cardiac index
CVP: Central venous pressure
HR: Heart rate
IAH: Intra-abdominal hypertension
IAP: Intra-abdominal pressure
ICU: Intensive care unit
MAP: Mean arterial pressure
MINORS: Methodological index for nonrandomized studies
P/F ratio: Ratio of partial pressure arterial oxygen and fraction of inspired oxygen
PCWP: Pulmonary capillary wedge pressure
PEEP: Positive end-expiratory pressure
PIP: Peak inspiratory pressure
SMD: Standardized mean difference
SOFA: Sequential Organ Failure Assessment
SVRI: Systemic vascular resistance index
UO: Urinary output
WSACS: Abdominal Compartment Society (formerly known as World Society of the Abdominal Compartment Syndrome)
